# Removable prosthodontics education and experience in the United Kingdom, from undergraduate to foundation training

**DOI:** 10.1038/s41415-025-8880-3

**Published:** 2025-10-10

**Authors:** Francesca Mullan, Helen Mather

**Affiliations:** School of Dental Sciences, Faculty of Medical Sciences, Newcastle University, Newcastle upon Tyne, United Kingdom

## Abstract

**Introduction** Concerns have been made previously that new graduates lack confidence and experience in removable prosthodontics.

**Aims** To explore undergraduate removable prosthodontic curricula and experience in UK dental schools, and removable prosthodontics experience and confidence of foundation dentists (FDs), in North East England.

**Methods** Two online anonymous questionnaires were sent via email.

**Results** Responses were received from 14 dental schools and 22 FDs. Expected thresholds averaged at four partial dentures and three complete dentures. FDs rated their undergraduate training as ‘good' or ‘very good' in complete and partial dentures, but ‘fair' to ‘very poor' for implant-supported overdentures. The number of dentures provided in foundation training (FT) averaged at six complete, 16 partial acrylic and two partial cobalt-chrome. FDs' confidence generally increased during FT in the provision of acrylic partial, complete and immediate dentures, whereas there was more variation and some decrease in confidence in cobalt-chrome partial dentures.

**Conclusion** Dental schools have employed strategies to ensure continued experience for their students. FDs thought highly of their undergraduate education in complete and partial dentures, but indicated improvement was needed for implant-supported dentures. During FT, confidence was reported in the provision of removable prostheses where they had gained the most experience.

## Key points

- Although more older adults are retaining some natural teeth, it is possible that more will require partial dentures, and providing complete dentures for the edentulous patient will become more complex. Removable prosthodontics education and experience remains a cornerstone of general dentistry.
- Dental schools are continuing to provide graduates with experience of removable prosthodontics to align with General Dental Council requirements.
- There is a recognised need to adopt digital technologies within undergraduate and postgraduate training to ensure graduates are prepared for the future of removable prosthodontics provision.

## Introduction

Changing trends in oral health of ageing populations in the United Kingdom (UK) have been observed for some time. Older adults are retaining their natural dentition, with edentulous populations dropping from 37% in 1968, to 6% in the combined populations of England, Wales, and Northern Ireland in 2009; although, nearly one in five adults were reported as wearing some form of partial removable denture.^1^ This change may indicate positive improvements in oral health and quality of life, but it also brings new complexities, such as becoming edentulous at an advanced age when it is more difficult to adapt to change, or increased desire for more complex dentistry to manage challenges of the partial dentition.^2^^,^^3^^,^^4^ While we await the results of the most recent Adult Dental Health Survey, the 2021 Census would suggest that the pattern of an ageing population is continuing.^5^

The General Dental Council (GDC) - the UK regulator - outlines the learning outcomes for undergraduate curricula, and includes the learning objective to ‘assess the need for, design, prescribe and provide biomechanically sound partial and complete dentures'.^6^ Dental schools however have autonomy in how they achieve these outcomes. Previous research has suggested variation among UK dental schools in the timing of removable prosthodontics teaching, differences in assessment of competency and experience numbers of removable prosthodontics cases carried out,^7^^,^^8^^,^^9^ expressing concern at the number of procedures, which averaged at 1-3 complete cases for compete dentures and three for partial dentures.

Wieder *et al*. (2013) suggested a regional variation in teaching hours dedicated to complete dentures, despite the number of required cases having more similarities; North and South were demarcated based upon an arbitrary line across the Midlands in England and included Scotland, Wales and Northern Ireland.^9^ Some schools, predominantly in the north, reported no issues with complete denture patient availability; however, this was not the case for all schools, particularly those in the south. On average, schools expected graduates to have completed 1-3 cases, a number that has declined over the years.^10^ Concerning removable partial dentures (RPDs), Clark *et al*. (2011) found variation in experience requirements, and where a target was declared, this was three RPDs, with a range of 3-9, where at least one RPD was of cobalt-chrome (CoCr) design.^10^ Although the experience in CoCr RPDs was relatively low, schools were making efforts to improve teaching overall, including simulated stages and videos of clinical demonstrations. The low reported experience in CoCr however was of concern for the authors. Generally, this does seem less removable prosthodontic experience compared with that reported by Murphy *et al*. (1972) when the average number of completed prostheses was 20, comprising 11 complete dentures, five partial dentures, two immediate dentures and two relines/rebases.^11^

It may be understandable that there will be regional variation in specific dental needs of populations surrounding dental schools, but, irrespective of this, new dental graduates should be able to meet the needs of any area within the UK. Those involved in foundation training (FT) have expressed disappointment in the undergraduate experience of removable prosthodontics and have been critical of what they perceive as variation among dental schools.^12^ New graduates will leave dental school most confident in those areas where they have gained most experience.^13^^,^^14^^,^^15^ However, dental schools and dental foundation training (DFT) providers have joint responsibility in ensuring that new dental graduates gain the experience needed to safely meet the dental needs of the diverse populations they will serve.

The aims of this study were to explore undergraduate removable prosthodontic curricula and experience in UK dental schools, and the experience and confidence of foundation dentists (FDs), in the North East of England, in removable prosthodontics.

## Methods

Ethical approval for the survey was granted by Newcastle University's Ethics Committee on 19 April 2023 (Ref: 2506/29958). Two questionnaires were developed: one specifically written for dental schools and the other for FDs. The dental school questionnaire enquired about the school's undergraduate removable prosthodontics curricula, including timing of delivery of content, specific clinical delivery, number of cases, any challenges in recruiting patients and use of simulation (Appendix 1). The questionnaire was sent in an invitation email to a sole individual identified as the coordinator (or equivalent) of removable prosthodontic teaching in each of the 16 UK dental schools with undergraduate programmes. A checkbox was included to confirm participants had read the information letter and gave their consent to participate. The FD questionnaire enquired about their reflections of the undergraduate teaching they received, further training in FT, experience in FT, specific clinical delivery in FT and changes in confidence during FT (linear numeric response 1-10; 1 = significant decrease in confidence and 10 = significant increase in confidence) (Appendix 2). Invitation emails were sent through a representative in the North East of England Deanery who was not involved in the research; 74 participants (50 FDs and 24 general professional trainees) were invited to complete the survey. General professional training is a two-year longitudinal programme combining FT in general practice and dental core training 1, with placements in restorative dentistry, paediatric dentistry and oral surgery in a secondary care setting. Email reminders were sent after two weeks and four weeks. Following closure of the form, data were exported from Microsoft Forms into a Microsoft Excel datasheet for analysis.

## Results

### Overview

**Fig. 1:**
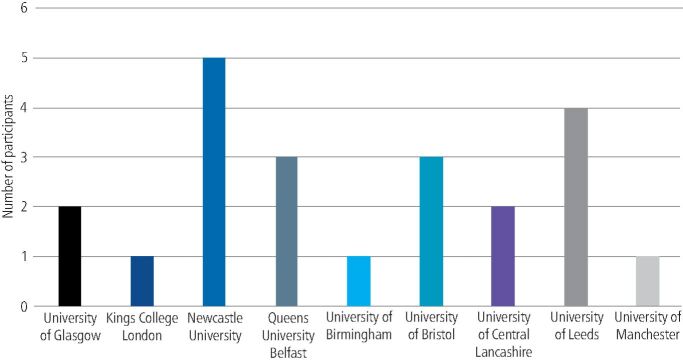
Graph displaying the number of FD respondents from each represented dental school

**Fig. 2:**
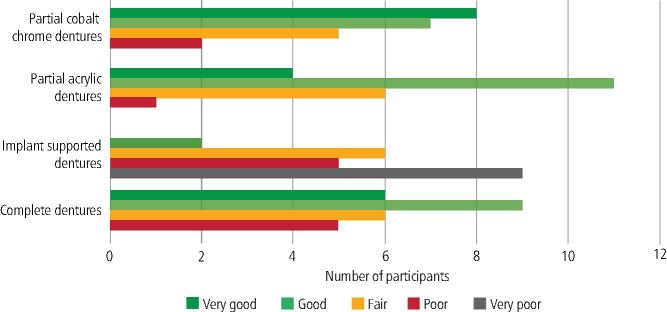
Graph displaying FDs' reflections of undergraduate removable prosthodontic teaching

In total, 14 dental schools and 22 FDs responded, resulting in an 87.5% and 29.7% response rate, respectively. Participants who responded to the FD questionnaire represented experience of removable prosthodontic education and training at nine out of 16 dental schools in the UK; the distribution is shown in Figure 1. FDs' reflections of their undergraduate teaching in removable prosthodontics was ‘good' to ‘very good' for complete dentures, CoCr partial dentures and acrylic partial dentures. However, they rated implant teaching to be ‘poor' to ‘very poor' (Fig. 2). All FDs reported taking part in a study day; 15 had tutorials with educational supervisors and 13 took part in case-based discussions.

### Complete dentures

Most (9 out of the 14) respondent dental schools commenced complete denture theoretical and practical teaching in third year. However, three schools commenced theory and practical teaching in second year, and one commenced teaching in fourth year. Most dental schools commenced provision of complete dentures on clinics in third year, with three dental schools commencing clinical procedures in fourth year. Copy denture techniques were taught by twelve of the respondent dental schools. Some schools had dedicated prosthodontic clinics, while others reported integrated restorative clinics; clinical staffing ratios ranged from 1:4 to 1:8.

Competency was reported to be assessed in differing ways, including feedback from clinical supervisors at each denture stage; formal assessments of competency in jaw registration and major (master) impressions; the student's own reflections on performance; and assessment of theory through written exams and via scenarios. Expected thresholds of complete dentures were reported by eight of the respondent dental schools, where thresholds ranged from one complete denture case to a total of six. Some schools combined expectations for complete and partial cases together. FDs' experience in the provision of complete dentures during FT ranged from 0-15.

Dental schools reported requiring 6-8 stages for the fabrication of a conventional complete denture, dependent on the speed of the student and appointment length. The stages included an examination, primary impressions, major impressions, jaw registration, try-in, fit, and a final review. In the FD responses, denture stages were reduced, with only four respondents completing all stages separately, two did not indicate that they record major impressions and 16 combined major impressions with the registration stage of complete denture construction.

### Partial dentures

Theoretical partial denture teaching commenced in third year in 11 of the respondent dental schools. Practical partial denture teaching commenced in third year in nine dental schools, three dental schools commenced it in second year, and one in fourth year. Competency in partial dentures was assessed in nine dental schools; methods included feedback from a supervisor at each clinical stage, assessment in written exams, summative assessment of jaw registration and CoCr denture design.

Expected threshold of number of partial dentures per student was reported in eight dental schools. This ranged from 2-5, commonly stipulating that at least one acrylic and one CoCr was included in this number. Experience in provision of acrylic partial dentures in FD ranged from 3-50 and a mean (SD) of 16 (10), whereas provision of CoCr dentures ranged from 1-4. Flexible dentures were reported as part of the curricula in two dental schools, but were offered in 7 of the 22 foundation practices in the North East of England.

Dental schools' reported stages for the provision of acrylic dentures were primary impressions, major impressions, definitive jaw registration, try-in, and delivery and review appointments. The number of appointments listed ranged from 5-8. For CoCr partial dentures, some schools included a primary jaw registration and a metal framework try-in stage. For denture designs, some schools scheduled time without the patient for students to survey the casts and finalise the denture design with a clinical supervisor. The number of expected appointments ranged from 6-8, with one stage being completed per appointment. The responses from FDs reported a reduced number of stages and appointments, with major impressions often combined with the registration stage, as reported by 14 FDs. However, five reported they did not record major impressions and one respondent indicated they would take major impressions if required and two indicated the registration stage would be completed only if required. There was also variation in the reported stages for CoCr partial dentures, commonly omitting the definitive registration; 68% of respondents provided a denture design but only one prescribed a path of insertion, with some responding this was not completed due to lack of a surveyor.

### Recruitment and simulation in dental schools

Difficulty in recruiting complete denture cases was reported by eight dental schools. To address this, a range of approaches to recruitment have been implemented, including lowering the expected threshold of complete denture output for students, encouraging self-referrals, referrals from general dental practitioners and review of previous patients. Some dental schools placed advertisements in pharmacies and local dental committees.

Simulation was used in ten dental schools and was used in both complete and partial denture stages. Analogue simulation with manikins was the most common type of simulation used for primary impressions, secondary impressions, tooth modifications and laboratory stages. Small group demonstrations on dental manikins were also used alongside online clinical observation videos.

### Further training and changes in confidence during foundation training

**Fig. 3:**
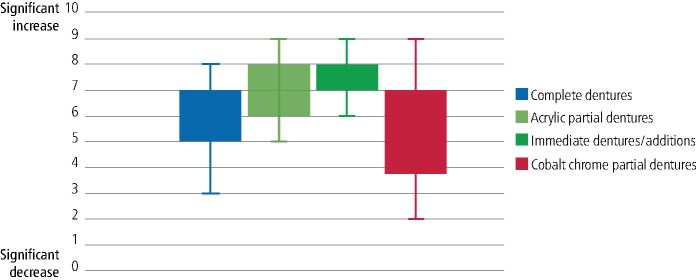
Reported change in confident of denture provision during FD, 1= significant decrease in confidence, 10=significant increase

Participants reported receiving a variety of further training in removable prosthodontics during FT, including one-to-one tutorials, study days and case-based discussions. Average number of dentures completed as an FD were: complete dentures = six (range 0-15); partial acrylic dentures = 16 (range 3-50); and partial CoCr dentures = two (range 1-4). When asked how their confidence in the provision of acrylic partial dentures changed during DFT, 20 reported increased confidence while two remained the same. Whereas, for CoCr partial dentures, six reported a decrease in confidence, four remained the same and 12 reported improvement. For complete dentures, 16 reported increased confidence, four remained the same and for two, confidence decreased. Reported changes in confidence are shown in Figure 3.

### Materials and equipment

Alginate was the most reported impression material used for primary impressions by dental schools. FDs either used alginate on its own or in combination with modelling plastic impression compound or sticks (e.g., greenstick). Alginate was also popular for major impressions and was reported by seven dental school responses and 19 FDs. The other materials reported were silicone and zinc oxide eugenol. Greenstick was commonly mentioned for border moulding or extending trays. Special trays were used by 19 FDs for their major impressions.

Facebow recording was taught in seven dental schools for jaw registration; however, it was acknowledged the technique was not routinely used clinically. All FDs reported regular use of wax knives (or equivalent) and Bunsen burners (or equivalent) whereas 14 used a Willis gauge for determining vertical dimension, 12 used a Fox's guide plane for occlusal plane assessment, ten used a hot plate (or equivalent), four used a copy box, two used intra-oral scanners and one used an Alma gauge for defining the position of the anterior central incisor teeth. No FDs used facebows or surveyors.

### Prosthodontic laboratory

There were seven dental schools which reported solely using their onsite prosthodontic laboratory, three that solely used an offsite facilities, and the remaining used a mix of both onsite and offsite, depending on the denture stage, such as casting metal framework, and referenced students completing their own denture laboratory stages in some cases. The laboratory turnaround ranged from 1-3 weeks between denture stages, with the most common being two weeks. Several responses indicated a longer time, such as three weeks for the metal framework. FDs reported turnaround times ranging from two days to four weeks, which was also dependent on the type of denture being produced and the stage of denture construction.

Only four dental schools reported using digital workflows in denture fabrication. Examples included 3D-printed special trays, CAD/CAM (computer-aided design/computer-aided manufacturing) metal framework and clasps.

## Discussion

Previous studies criticised undergraduate experience in removable prosthodontics and expressed concerns about a continued downward trend in experiential numbers.^1^^,^^7^^,^^9^ However, the responses in this study demonstrated that clinical case numbers were comparable with those reported ten and 17 years ago. General curricula and materials used on clinics have also remained comparable.

There has been a shift to an outcomes-based approach in dental education, where completion of competence-based assessments demonstrates achievement of skills considered essential attributes of the role.^16^ It was shown when one school implemented a new competence-based curriculum and students reported an increase in confidence in a range of clinical and non-clinical skills.^17^ However, concern has been expressed regarding the focus on competencies and the assessment methods used, as it is known assessment of competence is complex, requiring demonstrable longitudinal evidence of assimilation and application of multiple pieces of knowledge and skills simultaneously.^18^ Often, competence assessment includes a procedure-counting approach; however, it has recently been suggested that the number of procedures completed does not necessarily include the level of detail to provide assurances of graduate experience and therefore it is not recommended that such an approach is used in isolation to measure graduate competence.^19^ There remains to be an apparent focus on the number of procedures completed as a measure of experience, often in vocational trainer reflections of their own undergraduate experiences,^20^ without necessarily acknowledging the changes in the pedagogical approach in dental education since that time and the context of provision of undergraduate dental training. Although not all schools in this study recorded expected thresholds of cases, the majority of those that did not monitor experiential quotas monitored their students' capabilities by assessing competency in one or more stages of denture provision. Where there were reported challenges in recruitment of ‘suitable' complete denture cases, various measures were adopted, including simulated learning experiences, in order to ensure graduate experience in removable prosthodontics is maintained. This suggests dental schools continue to place value in removable prosthodontics and are taking measures to ensure undergraduate experience is in line with GDC requirements.^6^

This study was able to explore the experience of newly qualified dentists in the North East of England and identified that confidence improved in all removable procedure types during FT. The response rate, while lower than we would have preferred, is comparable with similar research.^12^ It would perhaps be anticipated in a regional study that the responses would be skewed towards North East of England graduates; however, there was at least some representation from other areas of the UK. The number of respondents for each area and therefore the results must be interpreted cautiously. It is perhaps unsurprising that FDs reported most improved confidence with procedures that they have been carrying out the most in FT: acrylic partial and immediate dentures. Responses to change in confidence with partial CoCr dentures showed greatest variation, with six respondents reporting a decline in confidence. This is in contrast to reflections of their undergraduate teaching, which were mostly positive for CoCr dentures. However, the results are consistent with previous research which identified that newly qualified dentists were providing less CoCr partial dentures compared with their undergraduate experience.^21^ Where dental schools reported thresholds for number of procedures of partial dentures, it was commonly stipulating that at least one acrylic and CoCr was included in this number.

Choice of material used for a denture design should be a shared decision with the patient that best serves their clinical needs and personal preferences. However, there are many factors that influence these decisions, including cost and profit margins for practices in relation to the fee structures from healthcare systems.^22^^,^^23^ As the respondents in this study are salaried FDs without out-of-pocket expenses for lab costs, one would hope that this was not an influence in decision-making. Previous research has suggested that new graduates were actively discouraged by practices to undertake CoCr partial dentures and struggled with partial denture design and its effective communication to laboratories.^21^ In our study, most reported providing denture designs; however, only one reported prescribing the path of insertion. A common reason provided for not prescribing designs was the lack of surveyors. Dental schools are actively trying to ensure that graduates leave with the clinical ability to prescribe partial denture designs using formative assessment of CoCr denture design to assess competency. Perhaps better communication and partnership with dental schools and FT in further training and education would help bridge the gap.

There are other differences between the dental schools' responses and those from FD that suggest there may be a disconnect early in dentists' careers. FDs tended to shorten or combine denture stages compared to what they had been taught at dental school. While an experienced clinician will develop their skills to effectively do this, that will not necessarily be true for a new graduate with limited experience, and might suggest haste rather than maximising clinical opportunity to hone and develop clinical skills. Time pressure on FDs may relate to the high demand in general practice, as increasingly fewer practices feel able to offer NHS (National Health Service) treatment, which increases the demand on those that do.^24^ Dental schools teach the traditional gold standard approaches to removable prosthodontics, recognising the transferrable skills in prosthodontics and occlusion. Newer trends in flexible dentures were not commonly taught (two dental schools) and digital workflows are currently limited to four dental schools and laboratory procedures only. While a limited number of FDs reported using digital workflows or providing flexible dentures, the responses were disproportionally higher. This provides some concern, particularly in relation to flexible dentures. Flexible dentures may be an appropriate prosthesis in a specific situation, but they are controversial due to their potential impact upon periodontal health.^25^^,^^26^ Therefore, if FDs are making the clinical decision to provide these prostheses, they must have sufficient skills to do so optimally. Targeted postgraduate training is required or there is a need for undergraduate programmes to adapt to changing methods of removable prosthodontic provision. A recent study in America looked into the use of CAD/CAM for complete and partial dentures in dental school predoctoral and advanced graduate prosthodontic education.^27^ Within the predoctoral teaching, CAD/CAM complete dentures were taught in 54.2% of courses and CAD/CAM partial dentures were taught in 37.5%.^27^ This suggests that although their use is more integrated than in UK education providers, it is likely still below that of use in private practice.

FDs rated their undergraduate teaching in implant-retained overdentures poorest. The GDC requires graduates to ‘recognise and explain to patients the range of implant treatment options, their impact, outcomes, limitations and risks'.^6^ While this does not extend to the provision of implant-supported mandibular overdentures (ISMODs) at the point of completion of training, it would be expected further postgraduate training be undertaken. However, it has been over 20 years since the McGill consensus and it would seem from this study that the UK is lagging behind on the provision and education of implant-retained overdentures, despite the acknowledged patient benefits.^28^^,^^29^^,^^30^^,^^31^ It is known that restoration of the edentulous mandible with two implants correlates to improvement in patient satisfaction and their oral health-related quality of life.^32^^,^^33^^,^^34^^,^^35^^,^^36^ It has also been suggested graduates have the transferable skills to be able to provide ISMODs, and undergraduates are capable of providing ISMODs that confer a similar level of patient satisfaction as those provided by specialists, with a recent review suggesting ‘consideration be given to greater incorporation into existing prosthodontic teaching of the constructions of ISODs'.^37^ Despite the FDs reflecting poor satisfaction of undergraduate teaching of implants, they did not mention any further teaching during FT. Continued learning and skills development are important, as confidence tends to decrease with those procedures with least familiarity, as shown with the decrease in confidence in the provision of partial CoCr dentures.

Dental schools reported challenges in recruiting prosthodontic cases. It was not within the scope of this study to determine if dental schools are finding it challenging to recruit patients more generally or that the challenge is more specific to removable prosthodontics. With current difficulties faced by the public accessing NHS dental services, it may be expected that dental schools would experience increased demand. However, dental school campuses are often not located in the most convenient or accessible locations for patients with comorbidities, which are often experienced by the removable prosthodontic patient.^38^ Furthermore, with older adults retaining their natural teeth for longer, there are fewer complete cases generally, and those cases are often considered more challenging and may not be deemed suitable for undergraduate training. Dental schools demonstrated interest in simulation and currently favoured the use of analogue simulation within their education programmes. Simulation using manikins is certainly not a novel concept within dental education and is universally integrated within pre-clinical and early clinical training. However, simulation is not a replacement for patient experience, and dental schools were aware of this and were trying to overcome difficulties in patient recruitment. Newer concepts of digital simulation were not reported for removable prosthodontics. There is evidence to suggest that these may be attractive to dental students; however, they may not provide any additional educational value over the traditional methods that are currently used.^39^

## Conclusion

The continued increase in older populations in the UK highlights the need for removable prosthodontic skills. Despite previous concerns, dental schools are continuing to maintain undergraduate experience in removable prosthodontics. DFT in the North East of England is continuing this education. Dental schools currently favour a traditional approach to removable prosthodontics curricula but were cognisant of future development needs in patient recruitment and simulation. New graduates valued their undergraduate education in conventional removable prosthodontics yet demonstrated independence from teaching by modifying prosthodontic stages early in their careers. New graduates would value more teaching in implant-retained prostheses. Digital workflows are not currently routinely used for removable prosthodontics in dental schools or DFT practices but this may represent future developments in undergraduate curricula as these technologies become embedded in removable prosthodontics practice. Furthermore, the responses indicated room for improvement in education of implant-retained removable prosthesis. It is firstly important that conventional fundamental prosthodontic principles are learnt and then applied to implant-supported prosthesis through continued lifelong learning.

**Table 1:** Appendix 1 Dental school questionnaire which enquired about the school's undergraduate removable prosthodontics curricula, including timing of delivery of content, specific clinical delivery, number of cases, any challenges in recruiting patients and use of simulation

I confirm I have read the information letter and consent to participate in this study. I understand the purpose and nature of this study and I am participating voluntarily. I understand that I can withdraw from the study at any time, without any penalty or consequences.				
On behalf of which dental school are you responding?				
In which year do your students complete the following?				
Do you teach copy denture techniques?	Yes		No	
Do you teach the use of facebow for jaw registration?	Yes	No		Other:
Is the altered cast technique taught or used for free end saddle(s) cobalt-chrome partial dentures?	Yes, taught	Yes, taught and used where indicated		No
Are flexible dentures taught or offered?	Yes		No	
Do you use simulation in your complete and/or partial denture teaching/training?	Complete dentures only	Partial dentures only	Both	Neither
If you use simulation in your complete and/or partial denture teaching/training, please provide further details:				
Is there an expected threshold of number of complete dentures per student?	Yes		No	
If yes to question above, what are your expected thresholds?				
Is competency assessed for complete dentures?	Yes		No	
If yes, please provide further details on assessment of competency for complete dentures				
Is there an expected threshold of number of partial dentures per student?	Yes		No	
If yes to question above, what are your expected thresholds?				
Is competency assessed for acrylic or cobalt-chrome partial dentures?				
If yes, please provide further details on how competency is assessed for partial dentures.				
Are dentures made as part of an integrated treatment plan?	Yes	No		Other:
In relation to the lab used for student prosthodontic work is it:	Onsite	Offsite		Other:
What is the staff-to-student ratio on the prosthodontics clinic?				
What is the lab turnaround time between stages?				
Are digital workflows used in complete or partial denture construction?	Complete dentures only	Partial dentures only	Both	Neither
If you answered positively to the previous question, please provide further information on the use of digital workflows				
Please list the clinical stages you teach for conventional complete dentures in order and indicate the average number of visits to complete each stage:				
What is the predominant impression material used for primary impressions?				
What is the predominant impression material used for major impressions?				
For jaw registration please indicate type of base most often used	Temporary full wax	Permanent		Other:
Do you have difficulty recruiting sufficient complete denture patients?	Yes		No	
Please outline how you recruit patients to address any shortfalls which may arise:				
Please list the clinical stages you teach in order and the average number of visits to complete each stage for acrylic partial dentures:				
What is the predominant material used for primary impressions for acrylic partial dentures?				
What is the predominant material used for major impressions for acrylic partial dentures?				
Please list the clinical stages you teach in order and the average number of visits to complete each stage for cobalt-chrome partial dentures				
What is the predominant material used for primary impressions for cobalt-chrome partial dentures?				
35. What is the predominant material used for major impressions for cobalt-chrome partial dentures?

**Table 2:** Appendix 2 Questionnaire for foundation dentists, enquiring about undergraduate reflections, further training in foundation training (FT), experience in FT, specific clinical delivery in FT and changes in confidence during FT (cont. on page 494)

I confirm I have read the information letter and consent to participate in this study. I understand the purpose and nature of this study and I am participating voluntarily. I understand that I can withdraw from the study at any time, without any penalty or consequences.																																				
Which university did you graduate from?																																				
How would you rate the quality of your undergraduate teaching on?	Complete dentures				Very poor				Poor							Fair				Good									Very good							
	Implant supported dentures				Very poor				Poor							Fair				Good									Very good							
	Partial acrylic dentures				Very poor				Poor							Fair				Good									Very good							
	Partial cobalt chrome dentures				Very poor				Poor							Fair				Good									Very good							
What teaching have you had regarding removable prosthodontics?	Tutorial with education supervisor							Study day						Case-based discussion									Other													
Please indicate what equipment you routinely use when undertaking removable prosthodontics.	Bunsen burner or equivalent			Wax knife				Fox's bite Plane			Willis Gauge				Alma Gauge			Hot plate or equivalent						Intraoral scanner						Surveyor				Facebow		Copy box
What is the average lab turnaround between stages?																																				
How many complete dentures have you provided as a foundation dentist (upper and lower are counted separately)?																																				
Please list the clinical stages you use for complete denture fabrication in order and indicate the appointment length for each stage:																																				
What is the predominant impression material you use for preliminary impressions for complete dentures?																																				
What is the predominant impression material you use for major impressions for complete dentures?																																				
Please indicate what type of tray is used for major impressions.	Stock tray											Special tray													Other											
How has your confidence regarding the provision of complete dentures changed over your foundation training?	1 Significant decrease	2				3				4			5				6				7					8					9					10 Significant increase
How many partial acrylic dentures have you provided as a foundation dentist (upper and lower are counted separately)?																																				
Please list the clinical stages you use for partial acrylic denture fabrication in order and indicate the appointment length for each stage.																																				
How has your confidence regarding the provision of partial acrylic dentures changed over your foundation training?	1 Significant decrease		2				3				4				5				6			7					8					9			10 Significant increase	
How has your confidence regarding the provision of immediate dentures/immediate denture additions changed over your foundation training?	1 Significant decrease		2				3				4				5				6			7					8					9			10 Significant increase	
Are flexible dentures offered at your FT practice?																																				
How many cobalt chrome partial dentures have you provided as a foundation dentist (upper and lower are counted separately)?																																				
Please list the clinical stages you use for cobalt chrome partial denture fabrication in order and indicate the appointment length for each stage.																																				
Do you provide partial denture designs to your lab for cobalt chrome dentures?	Yes																		No																	
Do you prescribe path of insertion/ path of displacement of cobalt chrome dentures to the lab?	Yes																		No																	
How has your confidence regarding the provision of partial cobalt chrome dentures changed over your foundation training?	1 Significant decrease		2				3				4				5				6				7					8					9			10 Significant increase
Do you have any comments regarding the differences between your undergraduate teaching of complete/partial removable prosthodontics and your experience in practice during your foundation year?																																				

## Data Availability

The datasets generated and/or analysed during the current study are available from the corresponding author on reasonable request.
